# Study on Electric Power Fittings Identification Method for Snake Inspection Robot Based on Non-Contact Inductive Coils

**DOI:** 10.3390/s25113562

**Published:** 2025-06-05

**Authors:** Zhiyong Yang, Jianguo Liu, Shengze Yang, Changjin Zhang

**Affiliations:** Hubei Key Laboratory of Modern Manufacture Quality Engineering, School of Mechanical Engineering, Hubei University of Technology, Wuhan 430068, China; liujianguo202306@163.com (J.L.); hbut87409049@163.com (S.Y.); 102310154@hbut.com (C.Z.)

**Keywords:** snake inspection robot, induction coil sensors, electric power fittings, BP neural network, genetic algorithm

## Abstract

In power inspection fields, snake-like robots are often used for transmission line inspection tasks, requiring accurate identification of various power fittings. However, traditional visual sensors perform poorly under varying light intensity and complex background conditions. This paper proposes a non-visual perception method for the high-precision classification of different power fittings (e.g., vibration dampers, suspension clamps, and tension clamps) in snake-like robot transmission line inspection for high-voltage lines. This method, unaffected by light intensity changes, uses machine learning to classify the magnetic induction electromotive force signals around the fittings. First, the Dodd–Deeds eddy current model is used to analyse the magnetic field changes around the transmission line fittings and determine the induction coil distribution. Then, the concept of condition number and singular value decomposition (SVD) are introduced to analyse the impact of detection position on classification accuracy, with optimal detection positions found using the particle swarm optimization algorithm. Finally, a BP neural network optimised by a genetic algorithm is used for power fitting identification. Experiments show that this method successfully identifies vibration dampers, tension clamps, suspension clamps, and transmission lines at detection distances of 5 cm, 10 cm, 15 cm, and 20 cm, with accuracies of 99.8%, 97.5%, 95.1%, and 92.5%, respectively.

## 1. Introduction

Snake robots with multiple degrees of freedom are capable of flexible movement in complex environments and are increasingly being considered an optimal choice for industrial transmission line inspection robots [1,2]. However, in order to achieve efficient and safe online motion, path planning and motion control must be performed according to different fixtures (damper, suspension clamp, and tension clamp). Therefore, the ability to identify the electric power fittings with high accuracy is a prerequisite for continuous and stable operation of the robot on the line [3].

At present, robots rely primarily on the visual method to identify the type of electric power fittings. This is typically achieved by utilising an on-board camera to capture a substantial number of images of electric power fittings, followed by the application of algorithms such as YOLO and R-CNN to differentiate between electric power fittings [4,5,6]. However, this method has certain limitations. Firstly, the accuracy of the visual recognition method is highly dependent on the resolution of the image. However, in the field environment, especially under the condition of insufficient light or strong light, it is easy to obtain low illumination images and overexposed images, which ultimately leads to a decline in the classification accuracy [7,8]. Furthermore, the image data contain not only the target electric power fittings but also a considerable amount of background noise, which increases the difficulty of classification and further reduces the reliability of recognition [9]. Additionally, the visual recognition method relies on deep learning algorithms, and the pre-training time is lengthy, which affects the real-time performance of the system [10]. Lidar is another common perception-based sensor, but its output of a sparse point cloud dataset presents a challenge to achieving the identification and classification of electric power fittings [11,12,13,14].

In addition to vision sensors, researchers have gradually begun to focus on the potential advantages of induction coil sensors [15,16] as a means of improving the adaptability of robots in blind-sighted environments. At present, the applications of induction coils can be broadly classified into two categories. The first category encompasses the installation of the coil on a wire inspection robot, which enables the robot’s position to be detected. By utilising the magnetic field surrounding the transmission line, Wu Gongping and colleagues [17] installed an induction coil array on the inspection robot’s double arms to detect the distance between the transmission line and the robotic arm, thereby enabling the robot to autonomously traverse obstacles. Similarly, F. Zorić and colleagues [18] developed an inspection robot. The work of automatically installing the gold fixtures based on magnetic localisation technology was conducted by J. Moore [19] and others. The induced electric potential data were collected and combined with a BP neural network to predict the distance between the UAV and the conductor. The aforementioned studies utilised the inverse characteristic of the magnetic field generated by the transmission line and the detection distance. The second category involves the installation of flexible induction coils on the operational joints of intelligent robots, with the objective of enhancing the robot’s flexible haptics. Xu Yizhuo et al. [20] employ a method of distinguishing the shape of objects by detecting changes in the voltage of induction coils on the skin of the fingers, which is then combined with machine learning. Li Ning et al. [21] utilise a data fusion strategy of friction electrical signals and electromagnetic induction signals to identify a wide range of objects. A variety of fruits have been successfully identified by Ma Zheng et al. [22] using a soft magneto-electric finger (SMF) comprising an array of liquid metal (LM) coils. This innovative approach enables self-supplied and multi-directional haptic sensing, facilitating the distinction between common objects with the aid of machine learning. Induction coil sensors are a prevalent technology in magnetic field environments, enhancing the intelligence of robots.

To better compare the characteristics of several robot environmental recognition sensors, their features are listed in Table 1.

This paper presents a method for distinguishing the types of power fixtures along a line using non-contact induction coil sensors, based on the aforementioned practical applications of induction coils. In conjunction with the initial application, it becomes evident that a stable magnetic field exists around the energised conductor, which can be further exploited [17]. When considered in conjunction with the second application, it becomes apparent that different shapes of power fixtures exert varying influences on the magnetic field. The field and the induced electromotive force (EMF) signals are collected using an array of induction coil sensors arranged in advance [21]. Finally, the machine learning method is used to learn and classify the EMF signals of different types of power fixtures. However, this method still requires resolution of the following issues:

How to construct a theoretical model of the magnetic field of electric power fittings, optimise the sensor layout using finite element analysis, and reduce the impact of the change in detection position on the accuracy of the final recognition results, in order to improve the recognition accuracy and shorten the training cycle of the machine learning algorithm.

In order to address the aforementioned issues and achieve contactless detection and classification of high-precision electric power fittings with inductive coil sensors, this paper proposes a multi-sensor information fusion method to complete the classification task of electric power fittings. This classification process comprises three stages: feature selection, feature extraction, and machine learning recognition [24]. The optimization of sensor detection positions is essentially a multi-variable and multi-constrained problem. The position of inductive coils is determined not only by the magnetic field variations around different fittings but is also influenced by factors such as the sensor’s shape, size, and the cross-sectional dimensions of the snake robot’s head joint. The particle swarm optimization (POS) does not rely on the strict mathematical properties of the objective function. As the magnetic field intensity around fittings cannot be precisely described by mathematical formulas, the PSO initializes particles to calculate fitness function values and updates particle positions iteratively to find optimal solutions, enhancing the accuracy of the optimization process [25]. Although BP neural networks can theoretically approximate any nonlinear relationship, they are prone to getting trapped in local optima during training, which affects classification performance [26]. In contrast, the genetic algorithm (GA) simulates biological evolution through selection, crossover, and mutation operations, enabling global search and avoiding the limitations of local optima [27]. Given that multiple inductive coil sensor signals need to be comprehensively compared to identify differences for classifying transmission line fittings, the GA is more suitable for this application. The specific contributions made in this paper are as follows:

(1) The magnetic field distribution model affected by the detection cross-section of the electric power fittings is established based on the Dodd–Deeds eddy current calculation model [28] and the cross-section characteristic function of the electric power fittings. A theoretical comparison of the distribution of magnetic field strength of the electric power fittings at the detection cross-section is to be made, with the trend of change in magnetic field strength and the extreme difference taken as the classification eigenvalue. This will enable the preliminary determination of the sensor arrangement and range.
(2) The concept of condition number, when combined with the singular value decomposition (SVD) method, is employed to analyse the impact of the detection position on the accuracy of the classification backpropagation results of electric power fixtures. This is followed by the use of an improved particle swarm algorithm. By means of an adaptive adjustment of the inertial weights, the optimal detection position matrix can be derived in order to reduce the impact of the detection position on the classification backpropagation results. The distribution of the induction coil sensor position can then be determined by combining this matrix with the structural characteristics of the serpentine robot.
(3) The trend of sensor signal change and the order of sensor signal extreme difference size are employed as the feature signals of a BP neural network fusion algorithm optimised by a genetic algorithm for the purpose of classifying inductive signals at the same detection position and completing the task of distinguishing electric power fittings.

**Figure 1:**
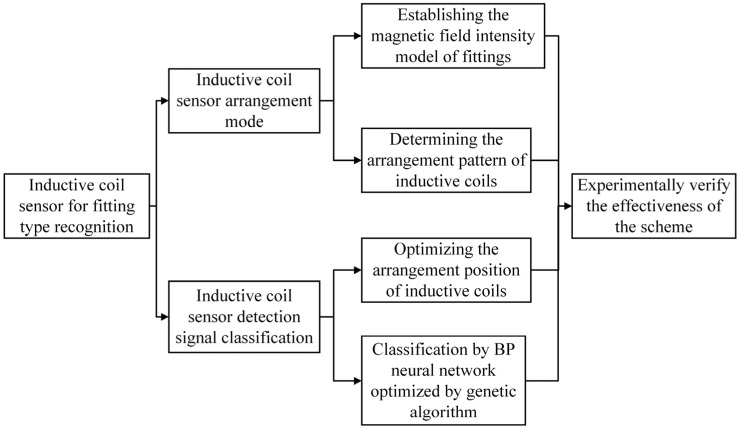
Key aspects of this paper.

The key aspects and processes of this paper are illustrated in Figure 1 to facilitate better understanding.

## 2. Snake Robot Operating Environment and Electric Power Fittings Recognition Mechanism

### 2.1. Subsection Robot Structure and Its Winding Motion Conditions

**Figure 2:**
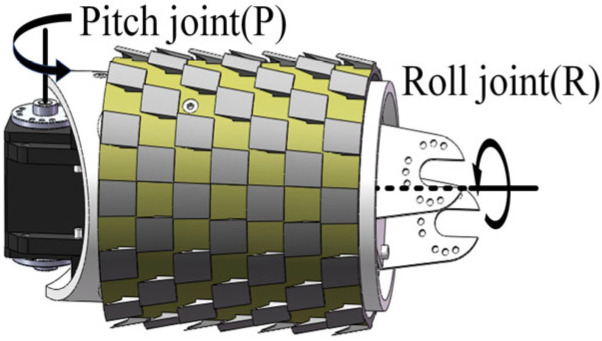
Snake robot unit.

As illustrated in Figure 2, the snake robot comprises ten identical units connected in series, with each unit exhibiting two degrees of freedom: pitch (P) and roll (R). Its motion is the result of the combination of the joints, which give rise to an equal-pitch spiral shape. The robot rolling joints undergo periodic rotation, with the friction between the outer sleeve and the wire serving as the primary means of support for its operation on the transmission line.

**Figure 3:**
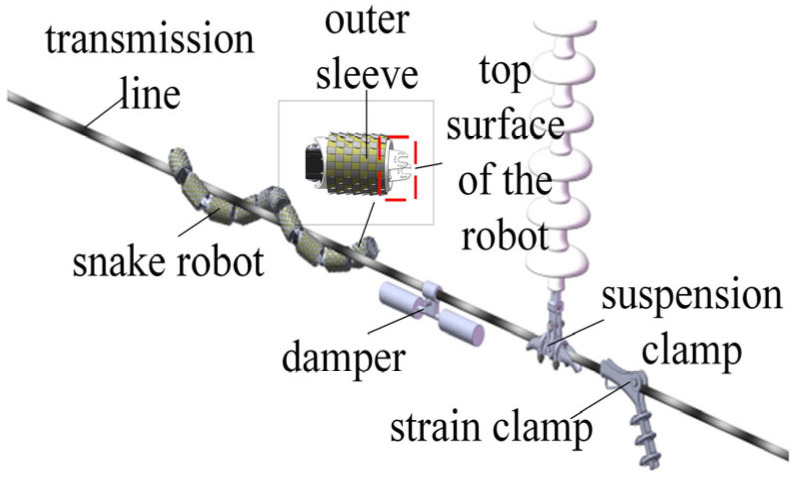
Operating environment of the snake robot.

As shown in Figure 3, the snake robot has two main operating states: (1) spiral rolling on the transmission line; (2) variable gait obstacle negotiation according to different electric power fittings. There are three common types of electric power fittings: dampers, suspension clamps, and tension clamps. When the snake robot runs on the transmission line, it needs to detect and distinguish the types of electric power fittings in real time through the inductive coil sensor array installed on the head joint of the snake robot. After determining the type, it will cross the barrier according to different gaits. Therefore, identifying the type of electric power fittings is a prerequisite for the smooth and continuous operation of the snake robot on the transmission line.

### 2.2. Mechanism for Identifying Types of Electric Power Fittings

**Figure 4:**
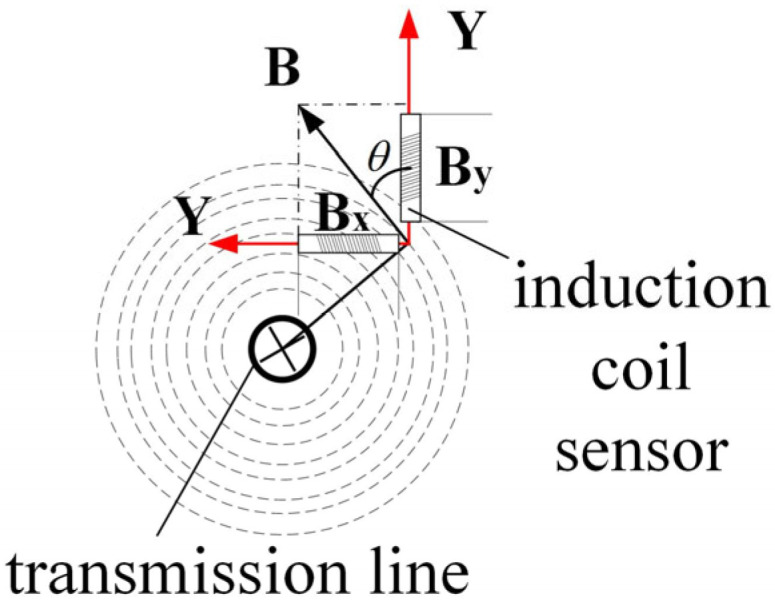
Detection of magnetic field intensity in X and Y components.

The configuration of the induction coil sensor is illustrated in Figure 4. In accordance with Faraday’s law of electromagnetic induction, a variation in the magnetic flux *ϕ* within a closed loop gives rise to an induced electromotive force *ε* in the coil, the magnitude of which is proportional to the rate of change of the magnetic flux.

(1) $$\varepsilon =-N\frac{d\phi }{dt}=-NS\cos\theta \frac{dB}{dt}$$

Furthermore, it can be derived from the Biot–Savart law:

(2) $$\varepsilon =-NS\cos\theta \cdot \mu_{0}\frac{dI}{2\pi rdt}$$

where *ε* is the coil-induced electromotive force, unit V; *B* is the magnetic induction intensity, unit T; *N* is the number of turns of the coil; *S* is the cross-sectional area of the coil; *θ* is the coil cross-section normal to the angle of the magnetic susceptibility, unit rad; *r* is the location of the point of the distance from the wire, unit m; *μ*_0_ is the vacuum permeability, unit N/A^2^; *dI*/*dt* is the rate of change in the current in the wire, unit A/s.

**Figure 5:**
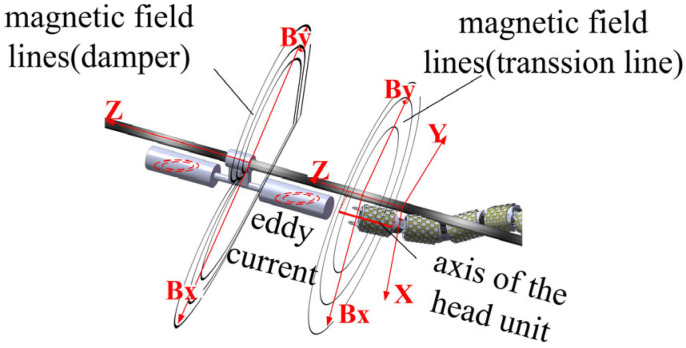
Mechanism of snake robot fitting identification.

As illustrated in Figure 5, the axis of the head rolling joint of the snake robot is maintained in a parallel orientation with the transmission line during operation. This configuration ensures that the front surface of the joint is perpendicular to the transmission line. Consequently, this study exclusively considers the X-Y plane of the magnetic field strength component. In the absence of electric power fittings, the magnetic field surrounding the transmission line is distributed in the form of concentric circles. However, the presence of electric power fittings induces eddy currents within the fittings, resulting in a distortion of the original magnetic field. This distortion is contingent upon the specific shape of the electric power fittings. In other words, the distribution of the magnetic field strength is affected by the presence of electric power fittings, resulting in variations in the size of the induction coil electric potential. According to this mechanism, Equation (1) can be qualitatively corrected.

(3) $$\left\{\begin{array}{l} \varepsilon_{x}=B_{x}(k)NS\\ \varepsilon_{y}=B_{y}(k)NS \end{array}\right.$$

where *B_x_*(*k*) and *B_y_*(*k*) are the components of the magnetic induction strength *B* in the X and Y directions, which can be detected by placing a one-dimensional induction coil sensor axis along the X and Y directions within the magnetic field, where *k* is a parameter of the type of electric power fittings; *N* is the number of turns of the magnetic sensor coil; and *S* is the magnetic sensor cross-sectional area.

## 3. Influence of Electric Power Fittings on the Magnetic Field of Transmission Lines

### 3.1. Modelling of Magnetic Field Strength Around Electric Power Fittings

Electric power fittings have complex shapes, and it is difficult to establish an accurate mathematical model; combined with the snake robot head unit detection characteristics, this paper uses the electric power fittings to detect the difference in the shape of the cross-section to replace the various fittings.

**Figure 6:**
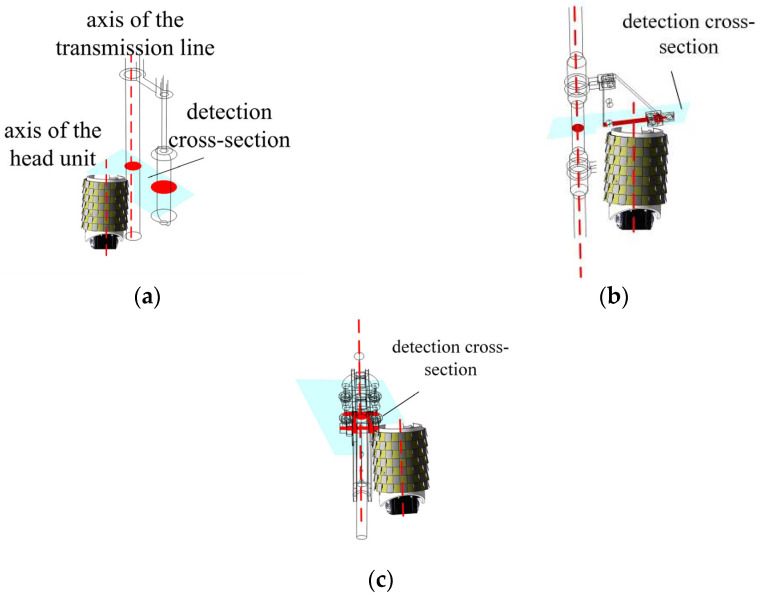
Detection cross-section of electric power fittings; (**a**) cross-section detection of damper; (**b**) cross-section detection of suspension clamp; (**c**) cross-section detection of tension clamp.

A snake robot head unit is installed on the front surface of the induction coil sensor for magnetic field detection, and an extension of this plane and electric power fittings intersection is realized, and the formation of the cross-section is the detection cross-section. Common electric power fittings comprise three types: anti-vibration hammer, suspension clamp, and tension clamp; according to the general shape of these fittings, the detection cross-section can be divided into the following shapes: Figure 6a anti-vibration hammer in the lower end of the transmission line is almost circular; Figure 6b suspension clamp in the upper end of the transmission line is almost rectangular; Figure 6c tension clamp in the transmission line presents a rectangle wrapped around the transmission line in the shape of the transmission line.

**Figure 7:**
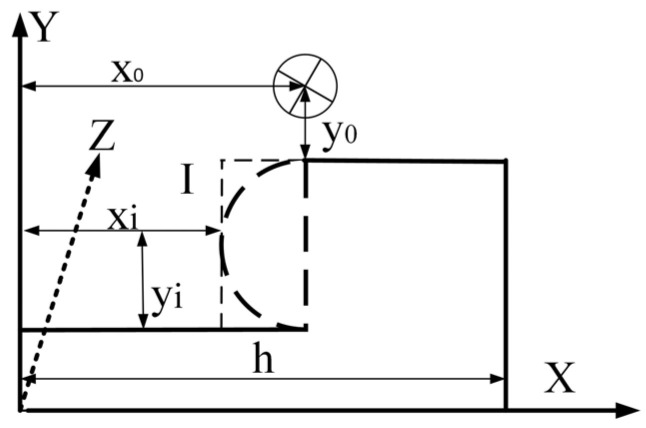
Magnetic field model around transmission lines influenced by electric power fittings.

To establish a simplified model of the magnetic field around the transmission line affected by electric power fittings as shown in Figure 7, the axis of the cylindrical transmission line is oriented along the *Z*-axis, and the direction of the current is also along the *Z*-axis; there is a flat plate with a length of h in the X-Y plane at a distance of *y*_0_ from the conductor, and the shape of the detected cross-sectional shape under the transmission line is represented by $K_{i}\left\{k_{i}(x_{i},y_{i})|(x_{i}<x_{0},y_{i}>0)\right\}$, where *k_i_* represents the parameter of the shape of the cross-section of the fixture and its position in the X-Y plane (where i = 1, 2, and 3, representing the circular cross-section, rectangular cross-section, and rectangular wound transmission line cross-section, respectively).

Since the magnetic field distortion causes eddy currents inside the gold fixture, combined with the classical Dodd–Deeds model, the main idea of solving the eddy current problem in this section is as follows: firstly, establish a system of Maxwell’s equations; then, combine the magnetic vector potential as an intermediate physical quantity; and finally, the magnetic vectors are deflected with respect to X and Y to derive the expressions of the *B_x_* and *B_y_* components. Therefore, the time-harmonic Maxwell’s system of equations for the low-frequency electromagnetic field is established first as follows:

(4) $$\left\{\begin{array}{l} \nabla \cdot H=J_{s}+J_{e}\\ \nabla \times E=-j\omega B\\ \nabla \cdot D=0\\ \nabla \times B=0 \end{array}\right.$$

where *H* is the magnetic induction; *J_s_* is the source current density; *J_e_* is the eddy current density; *E* is the electric field strength; *B* is the magnetic flux density; and *D* is the electric flux density. Directly solving the electromagnetic field of the eddy current problem is complicated and difficult; in order to simplify the calculation in the Dodd–Deeds model, the fluctuation equation of the magnetic vector potential *A* is usually used as an unknown function for describing the distribution of the magnetic field, and the expression is as follows:

(5) $$\left\{\begin{array}{l} \nabla \cdot A=0\\ \nabla \times A=B \end{array}\right.$$

Equation (5) describes and guarantees the uniqueness of the magnetic vector potential with respect to the magnetic field B. In order to obtain the final magnetic field distribution combined with Equation (4) the fluctuation equation of the magnetic vector potential A can be obtained:

(6) $$\nabla^{2}A=k^{2}A$$

where $k^{2}=j\omega \mu \sigma$; *w* is the current angular frequency; *μ* is the magnetic permeability; and *σ* is the electrical conductivity. In order to finally solve the magnetic vector potential results, the solution region is artificially restricted in the odoulidis method to satisfy the Dirichlet condition, and the final differential equations are obtained as a general solution of the magnetic vector potential, which, according to the simplified model, focuses only on the distribution of the magnetic field in the vicinity of the detection surface, and therefore, the magnetic vector potential *A* = 0 is defined for *x* = 0 and for *x* = *h*. The corrected induced electromotive force of Equation (3) is related to the strengths of the magnetic field *B_x_*, *B_y_* is related, and the expression for the magnetic vector potential *A* is defined as a function that depends only on x and y, respectively:

(7) A(x,y)=X(x)Y(y)

This can be obtained by bringing in Equation (7) and combining it with the separated variable method commonly used for solving ordinary differential equations:

(8) $$\left\{\begin{array}{l} A(x,y)=X(x)Y(y)\\ X(x_{i})=\sin(\alpha_{i}x)\\ Y(y_{i})=C_{i}e^{\lambda_{i}y}+D_{i}e^{-\lambda_{i}y} \end{array}\right.$$

where αi=iπ/h are the eigenvalues, $\lambda^{2}=k^{2}+{\alpha_{i}}^{2}$; *C_i_* and *D_i_* are the coefficients of the differential equations and are related to the specific cross-section shape. The components of the magnetic induction intensity in the *x* and *y* directions in the region of *I* are finally obtained, and in order to highlight the influence of the cross-section shape on the magnetic field, the coefficients of the differential equations are uniformly expressed in this paper as *k_i_* cross-section correction coefficients [28].

(9) $$\left\{\begin{array}{l} B_{x}=\frac{\partial A}{\partial y}=\sum_{i=1}^{\infty }\sin(\alpha_{i}x)e^{\lambda_{i}y}-\sum_{i=1}^{\infty }k_{i}\sin(\alpha_{i}x)e^{-\lambda_{i}y}\\ B_{y}=\frac{\partial A}{\partial x}=\sum_{i=1}^{\infty }\left[\alpha_{i}\cos(\alpha_{i}x)+\sin(\alpha_{i}x)\right]e^{\lambda_{i}y}+\sum_{i=1}^{\infty }k_{i}\left[\alpha_{i}\cos(\alpha_{i}x)+\sin(\alpha_{i}x)\right]e^{-\lambda_{i}y} \end{array}\right.$$

The magnetic field strength, *B_x_* and *B_y_*, is composed of two parts, as can be seen in the Formula (9). The former part of the formula is related to the original cross-section shape function, while the latter part is related to the coefficient function *k_i_*, which is dependent on the shape function. The former part of the formula is related to the transmission line magnetic field, while the latter part is related to the detection of a change in the cross-section shape. The formula provides a theoretical basis for the non-contact induction coil method used to identify electric power fittings on a snake robot.

### 3.2. Trend of Magnetic Field Strength in the Detected Cross-Section

In accordance with Equation (9), the trends of the magnetic field strength (*B_x_* and *B_y_*) components in the detection plane of the vibration damper, suspension clamp, tension clamp, and transmission line are calculated in the X and Y directions, respectively. This is realized in order to facilitate comparison of the differences in the magnetic field strength resulting from the disparate detection cross-sections; the fitted trend lines and the magnitude of the polar deviation are employed to reflect the discrepancies in the alterations of the magnetic field strength components along the X and Y axes in the detection cross-sections of the diverse electric power fittings.

**Figure 8:**
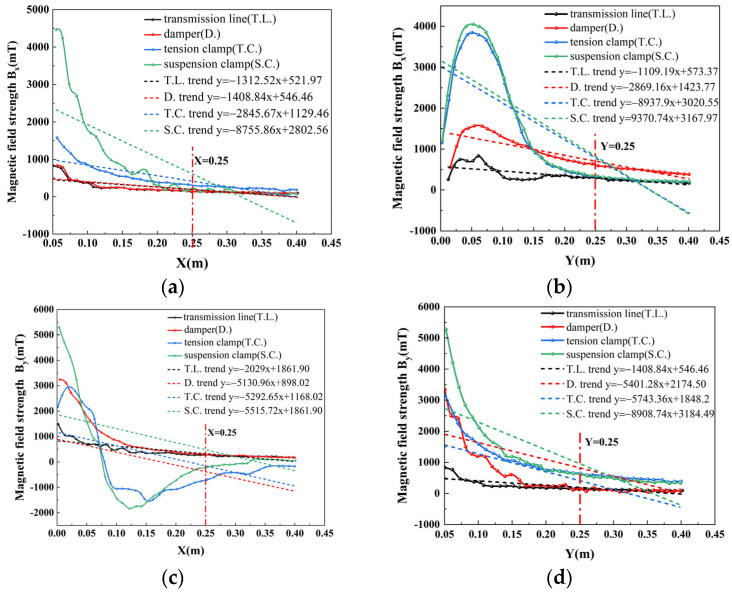
Variation trend of magnetic induction intensity in X-Y directions: (**a**) variation trend of *B_x_* in the X direction; (**b**) variation trend of *B_x_* in the Y direction; (**c**) variation trend of *B_y_* in the X direction; (**d**) variation trend of *B_y_* in the Y direction.

(1) Figure 8a illustrates that, within the range of 0.05 m to 0.25 m, a variety of electric power fittings exhibit a magnetic field strength that aligns with the observed trend. As the distance increases, the magnetic field strength also rises. The strength of the magnetic field decreases in comparison to the slope of the trend line. It can be observed that tension clips and suspension clips undergo the most significant amplitude change, whereas the transmission line and damper exhibit a comparatively minor amplitude change. The latter two variables are difficult to distinguish from one another.
(2) As illustrated in Figure 8b, within the range of 0 m to 0.25 m, the variation in tension clamp and suspension clamp amplitudes is comparable, with the latter exhibiting the greatest amplitude. Conversely, the amplitude of the transmission line and damper is relatively minor, yet the gap is considerable, rendering the detection of changes in magnetic field strength (*B_x_*) along the *Y*-axis a straightforward process. This allows for the differentiation between the four aforementioned types.
(3) As illustrated in Figure 8c, within the range of 0.05 m to 0.25 m, the trend line slopes of the remaining electric power fittings, with the exception of transmission lines, exhibit a similar pattern. However, the trend curve slopes are more pronounced. A negative value of −5000 indicates that the change in magnetic field strength (*B_x_*) can be readily discerned by the induction coil. However, to obtain a more pronounced discrepancy in the detection data, it is necessary to increase the spacing between the coil detections.
(4) As shown in Figure 8d, in the range of 0 m to 0.25 m, the slopes of the trend lines of electric power fittings other than transmission lines are large, and the difference is large, and a smaller detection distance of the induction coils can detect a large difference in data.

The magnetic field strength of each electric power fittings decreases along the *X*-axis and *Y*-axis from 0 m as the distance increases. However, the area of greatest variation is concentrated within 0.25 m from the centre of the transmission line. In this range, the degree of change is reflected by the use of polar deviation.

(10) $$\Delta B=B_{\max}-B_{\min}$$

where ∆*B* is the pole difference; *B_max_* is the maximum magnetic induction; and *B_min_* is the minimum magnetic induction.

According to Table 2, it can be concluded that the magnitude of the polar deviation of *B_x_* and *B_y_* both in the X and Y directions follow an obvious pattern: ∆*B*_3_ > ∆*B*_4_ > ∆*B*_2_ > ∆*B*_1_ But comparing the variation of *B_x_* in the X direction, it can be seen that ∆*B_3x_* > ∆*B*_4*x*_ > ∆*B*_2*x*_ ≈ ∆*B*_1*x*_, which is so similar to the results reflected in the analysis of Figure 7 that it tends to be difficult to distinguish between transmission line and damper, so it is not necessary to arrange induction coil sensors along the X direction to detect the *B_x_* component. While comparing the polarity of *B_y_* along the X and Y directions reveals that the gap between ∆*B*_1_, ∆*B*_2_, ∆*B*_3_, and ∆*B*_4_ is the largest in the X direction, Figure 8c demonstrates that the slopes of the trend lines of the three fixtures, except for the transmission line, are large and yet the gap is very small, and thus the inductive coils through the sensors in the *B_y_* along the X direction should be arranged with a large spacing.

### 3.3. Influence of Electric Power Fittings on Magnetic Fields and Detection Methods

Why electric power fittings affect the magnetic field around the transmission line can be qualitatively explained by comparing the distribution of magnetic force lines with and without conductor fittings. Using comsol simulation software 6.2 for finite element analysis, finite element modelling parameters are consistent with the standard wire fixture standard, and the model and basic parameters are shown in Table 3.

#### 3.3.1. Influence of Damper on Magnetic Field Distribution Around Transmission Lines and Detection Methods

**Figure 9:**
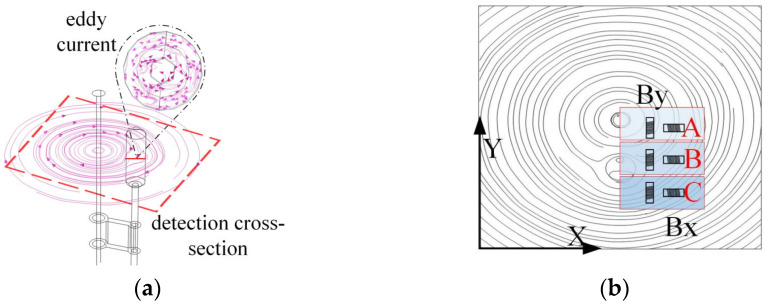
Impact of damper on magnetic field and detection method: (**a**) spatial magnetic field line distribution; (**b**) magnetic field line distribution at detection cross-section.

As illustrated in Figure 9a, the eddy currents generated within the damper impede the magnetic field surrounding the transmission line. As illustrated in Figure 9b, the damper induces a deflection in the magnetic field lines surrounding the transmission line, resulting in the formation of a magnetic field distribution analogous to that of an inverted ‘water droplet’. This phenomenon causes the original circular magnetic field lines of the transmission line in area B to gradually diminish in size, directing them towards area C, which is situated directly beneath the damper.

This inverted drop-shaped magnetic field distortion facilitates the detection of the By component. The *B_y_* and *B_x_* components can be detected simultaneously by placing the induction coil sensor in the position shown in Figure 9b. From region A to region C, the magnetic lines of inductance gradually contract, resulting in an increasing number of magnetic lines of inductance passing through the By induction coil sensor, while the angle between the magnetic lines of inductance and the sensor cross-section gradually decreases. At the same time, the angle between the magnetic lines passing through the *B_x_* induction coil sensor and the sensor cross-section decreases, but the density of magnetic lines in the sensor cross-section increases accordingly. As a result, the values of *B_y_* and *B_x_* gradually increase from region A to region C, i.e., near the damper, and there is a significant increase in the detected values compared to those near the straight wire alone.

#### 3.3.2. Effect of Suspension Clamps on Magnetic Field Distribution Around Transmission Lines and Detection Methods

**Figure 10:**
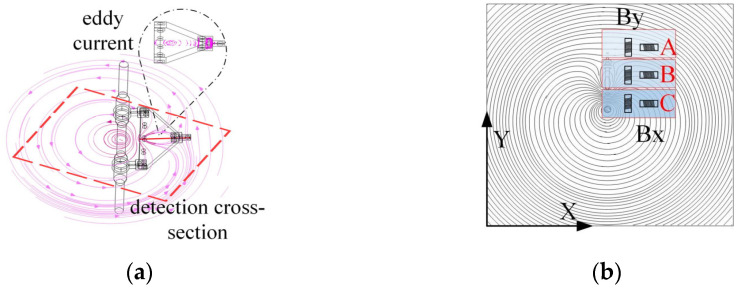
Impact of suspension clamp on magnetic field and detection method: (**a**) spatial magnetic field line distribution; (**b**) magnetic field line distribution at detection cross-section.

As shown in Figure 10a, the eddy currents generated inside the suspension clamp’s connecting plate affect the original circular magnetic field lines. Figure 10b further illustrates this effect, where magnetic field lines near the suspension clamp are contracted, resulting in a horseshoe-shaped distribution. This causes the magnetic flux to gradually concentrate from region A towards the connecting plate and conductor between regions B and C.

The distortion of the horseshoe-shaped magnetic field lines facilitates the detection of the *B_y_* and *B_x_* components. During the detection of the suspension clamp, inductive coil sensors are installed as shown in the figure to measure the *B_y_* and *B_x_* values. As the movement progresses from region A to region C, the magnetic flux lines become progressively denser, leading to an increase in the number of flux lines passing through the *B_y_* and *B_x_* sensor coils. The angle between the flux lines and the sensor also decreases, and some lines even pass perpendicularly through the sensor coils. Consequently, the *B_y_* and *B_x_* values from region A to region C, i.e., near the suspension clamp, significantly increase. These values are not only higher than those detected near a straight conductor but also exceed those measured near a damper.

#### 3.3.3. Impact of the Tension Clamp on the Magnetic Field Distribution Around Transmission Lines and Detection Methods

**Figure 11:**
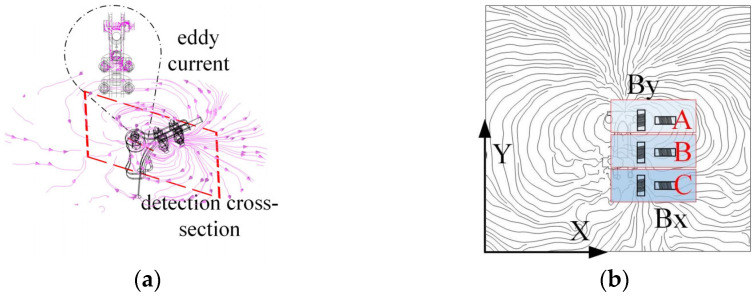
Impact of Tension Clamp on magnetic field and detection method: (**a**) spatial magnetic field line distribution; (**b**) magnetic field line distribution at detection cross-section.

As shown in Figure 11a, the double-sided clamp plates of the tension clamp generate internal eddy currents, altering the shape of the magnetic field lines. Figure 11b shows the contraction of magnetic field lines near the tension clamp, forming a pattern similar to the “infinity” symbol. This causes the magnetic flux lines to contract at both the upper and lower parts of the clamp at the detection cross-section, especially as the clamp plates converge from region B to region C.

The “infinity symbol” distortion of the magnetic field facilitates the detection of the *B_y_* and *B_x_* components. During the detection of the tension clamp, inductive coil sensors are installed as shown in the figure to measure the *B_y_* and *B_x_* values. In region A, the magnetic flux lines form a ring-like shape, which is favourable for detecting *B_y_*. In regions B and C, the magnetic flux lines contract, especially in region C, where the angle between the flux lines and the *B_y_* and *B_x_* sensor coils decreases, and the flux density increases. Therefore, from region A to region C, the *B_y_* and *B_x_* values near the tension clamp gradually increase. These values are not only higher than those detected near a straight conductor but also exceed those measured near damper attachments. Furthermore, due to the broad extent of the “infinity symbol” shaped distortion, its influence is larger than that caused by the damper and suspension clamp.

## 4. Distribution of Inductive Coil Sensor Array and Electric Power Fittings Classification Algorithm

### 4.1. Uncertainty Analysis of Fittings Type Recognition

The process of inferring the type of electric power fittings from the magnitude of induced electromotive force (EMF) is a typical example of a “cause-and-effect” problem. The key factor affecting the accuracy of the inference in this paper is the rational distribution of the inductive coil sensor array. The mathematical expression for inferring the type of electric power fittings from the induced EMF can be written as follows:

(11) KI=Pε

In the equation, *P* is the detection matrix determined by the detection coordinates; *KI* is the type of fitting; *ε* is the induced electromotive force.

In practical measurements, disturbances in sensor position measurement errors can cause significant fluctuations in the inference results. This paper introduces the concept of singular value decomposition (SVD) to reflect the sensitivity of the inference to small variations in the input data. From Equation (11), it is known that when matrix *P* is nonsingular, the difference ∆*ε* causes a disturbance ∆*KI* in *KI.*

(12) P(Δε+ε)=KI+ΔKI

Based on the definition of the operator norm, the relative error inequality can be derived as follows:

(13) $$\frac{\left\|\Delta \varepsilon \right\|}{\left\|\varepsilon \right\|}\leq \left\|P\right\|\cdot \left\|P^{-}\right\|\frac{\left\|\Delta KI\right\|}{\left\|KI\right\|}$$

(14) $$\frac{\left\|\Delta \varepsilon \right\|}{\left\|\varepsilon \right\|}\leq \mathrm{co}nd(P)\frac{\left\|\Delta KI\right\|}{\left\|KI\right\|}$$

In the equation, *cond*(*P*) represents the condition number. To calculate *cond*(*P*), the SVD representation of the detection matrix *P* is given as follows:

(15) $$P=UDV^{T}$$

In the equation, *U_m_*_×_*_m_* and *V_n_*_×_*_n_* are orthogonal matrices, and *D_m_*_×_*_n_* is a diagonal matrix whose diagonal elements are the singular values of *P*, *0* ≤ *δ*_1_ ≤ *δ*_2_ ≤ *δ*_3_ ≤ … ≤ *δ_r_* (where *r* is the rank of matrix *P*). The condition number of the detection matrix *cond*(*P*) can be determined by the ratio of the largest singular value to the smallest singular value:

(16) $$cond(P)=\frac{\delta_{r}}{\delta_{1}}$$

The condition number reflects the degree of variation in the scaling effect of matrix *P* in different directions. If *cond*(*P*) is too large, it indicates that matrix *P* is ill-conditioned and highly sensitive to small fluctuations. Conversely, if *cond*(*P*) approaches 1, it indicates that matrix *P* is well-conditioned and insensitive to disturbances. From Equation (16), it can be seen that the detection position of the coil inductive sensor can effectively reduce the interference of measurement disturbances on the final inferred fitting type results.

### 4.2. Optimization of Inductive Coil Sensor Distribution

In recent years, the particle swarm algorithm, based on a global random search approach, continuously updates position and velocity information during iterative processes, according to both the particle’s own position and the global optimum. Based on the analysis in Section 4.1, the detection position of the inductive coil sensors can affect the accuracy of the final inference. To reduce measurement interference by adjusting the sensor distribution, this paper employs an improved particle swarm algorithm with inertia weight adaptation to find the optimal sensor distribution method.

Based on previous research, the fitness function is defined as follows:

(17) $$FinFun=\left|cond(P)-1\right|$$

Here, *P* is the detection position matrix, with each element representing the coordinates of the detection positions [29]:

(18) $$\begin{array}{l} X_{m}=\left[x_{m1},\cdots ,x_{mn}\cdots ,x_{mN}\right]\\ Y_{m}=\left[y_{m1},\cdots ,y_{mn}\cdots ,y_{mN}\right] \end{array}$$

In this context, *n* presents the order of the measurement points, *N* refers to the number of measurement points and the dimensionality of the objective search space in the optimization algorithm, *m* denotes the order of the particles, and *M* represents the number of particles. Based on the analysis in Section 4.1, the detection position matrix *P* is considered well-conditioned only when *cond*(*P*) approaches 1. Therefore, the optimization objective is as follows:

(19) Minmize(FitFun)

Based on the analysis in Section 3.1, the magnetic field intensity differences of the electric power fittings are primarily concentrated within the range of (0, 0.25), while the detection area of the snake robot’s head joint is located only on one side of the electric power fittings. Therefore, the optimization region is as follows:

(20) $$\left\{\begin{array}{l} 0m<x<0.25m\\ 0m<y<0.25m \end{array}\right.$$

The particle velocity update formula is as follows:

(21) $$\left\{\begin{array}{l} V_{x,m}^{t+1}=\omega V_{x,m}^{t}+c_{1}r_{x,1}^{t+1}(X_{Hbest,m}-X_{m}^{t})+c_{2}r_{x,2}^{t+1}(X_{Gbest,m}-X_{m}^{t})\\ V_{y,m}^{t+1}=\omega V_{y,m}^{t}+c_{1}r_{y,1}^{t+1}(Y_{Hbest,m}-Y_{m}^{t})+c_{2}r_{y,2}^{t+1}(Y_{Gbest,m}-Y_{m}^{t}) \end{array}\right.$$

In the equation, *w* represents the inertia weight; *c*_1_ and *c*_2_ are the learning factors; *r* is a random number within the range (0,1). The particle position update equation is as follows:

(22) $$\begin{array}{l} X_{m}^{t+1}=X_{m}^{t}+V_{x,m}^{t+1}\\ Y_{m}^{t+1}=Y_{m}^{t}+V_{y,m}^{t+1} \end{array}$$

The weight adjustment strategy is as follows:

(23) $$\omega^{t}=(\omega_{start}-\omega_{end}){\left(\frac{T-1}{T}\right)}^{2}+\omega_{end}$$

**Figure 12:**
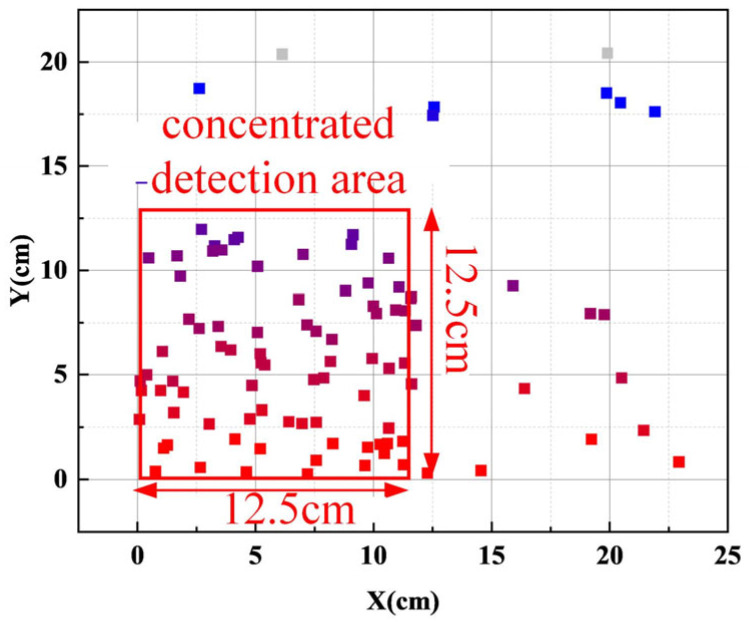
Optimal detection points map.

Here, *w_start_* is the initial weight; *w_end_* is the termination weight at the end of iteration; *t* is the current iteration count; and *T* is the maximum iteration time. The initial inertia weight is set to 0.9, the final inertia weight is 0.4, and the learning factors *c*_1_ = *c*_2_ = 1.5, leading to the optimal detection point map shown in Figure 12.

Since the optimal detection points are concentrated within a square area with a side length of 12.5 cm, they are uniformly distributed within this region. Additionally, due to the size of the inductive coil sensors, it is not feasible to place a sensor at every point. To accommodate the cylindrical structure of the snake robot’s head unit, the detection area is defined as a circular cross-section with a diameter of 12.5 cm. The detection locations are arranged to cover multiple optimal detection points, with the sensor spacing corresponding to the average distance between monitoring points in the concentrated detection area. Additionally, based on the analysis in Section 2 and the characteristics of the detection methods for each fitting, the sensors are arranged in the X and Y directions for *B_x_* and *B_y_*. Therefore, the sensor distribution is arranged as shown in Figure 12. For the *B_y_* detection, the inductive coils are spaced 1.5 cm and 3 cm in the X direction and 1 cm in the Y direction. For the *B_x_* detection, the inductive coils are spaced 1.5 cm in the X direction and 2 cm in the Y direction. The inductive coil sensors are cylindrical, with a length of 2.2 cm and a diameter of 0.8 cm.

**Figure 13:**
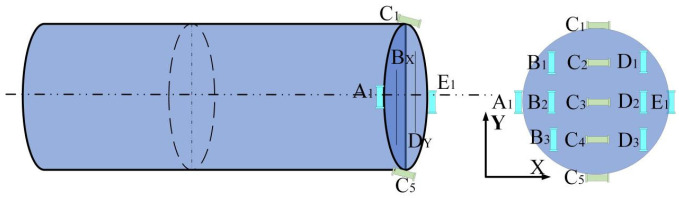
Distribution of inductive coil sensors.

The distribution of the inductive coil sensors on the snake robot’s head joint is shown in Figure 13. To accommodate the robot’s cylindrical head joint, the overall design uses a hollow cylindrical sleeve, facilitating installation and preventing interference with the movement of other joints. The end face is equipped with 13 cylindrical inductive coil sensors, named A_1_, B_i_, C_i_, D_i_ and E_1_ (where *i* = 1, 2, 3, 4, 5). The axes of the A_1_, B_i_, C_i_, D_i_, and E_1_ series sensors are parallel to the *Y*-axis, mainly used to detect the variation trend of the magnetic flux density *B_y_* along the *Y*-axis. The axes of the C_i_ series sensors are parallel to the *X*-axis, detecting the variation trend of the magnetic flux density *B_x_* along the *Y*-axis. Furthermore, the A_1_, B_i_, D_i_, and E_1_ series sensors can be used in combination to detect the variation trend of the magnetic flux density *B_y_* along the *X*-axis.

### 4.3. Induced Electromotive Force (EMF) Signal Classification Algorithm

The classification of transmission lines, dampers, suspension clamps, and tension clamps based on the voltage signal variations from the 13 inductive coil sensors falls within the scope of multi-source information fusion. The key challenge lies in feature signal selection and extraction, followed by training with a computer to achieve autonomous classification. This project, based on the previously studied electromagnetic field intensity differences around the fittings and the designed sensor distribution, aims to use the specific variation trends of sensor signals as feature signals to achieve classification. The autonomous classification is then performed using a genetic algorithm-optimised backpropagation neural network.

**Figure 14:**
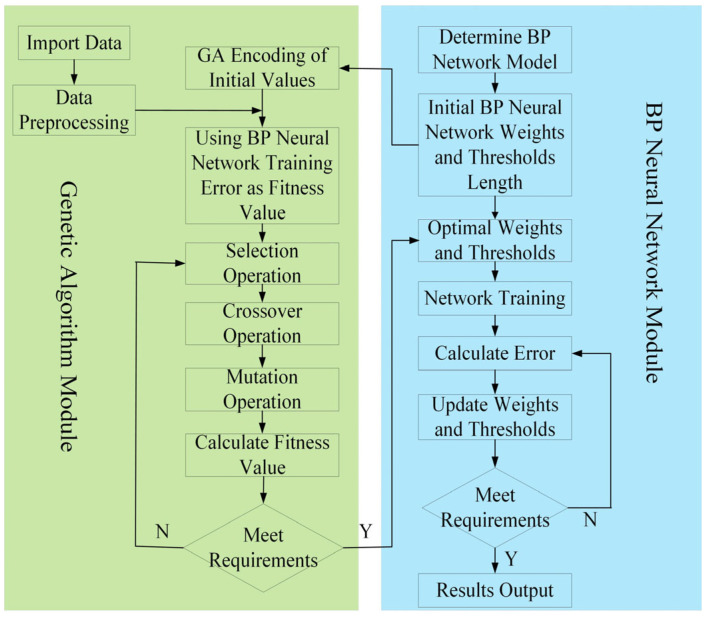
Flowchart of GA-optimised BP neural network algorithm.

The process of optimizing the weights and thresholds of the BP neural network using a genetic algorithm is shown in Figure 14. In this process, the weights and thresholds of the BP neural network are encoded as a biological population. The fitness of each individual is calculated using a fitness function, followed by genetic algorithm operations such as selection, crossover, and mutation. The optimised results are then applied to the neural network’s weights and thresholds, improving the predictive accuracy of the network’s training.

**Figure 15:**
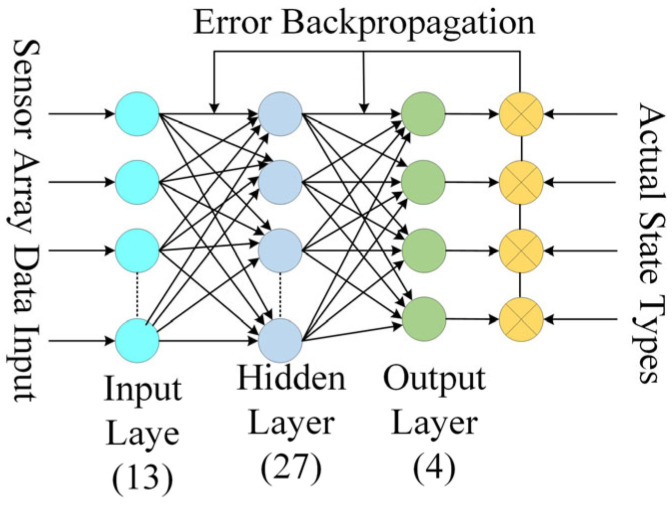
Neural network model.

Considering that there are 13 sensors used for feature signal acquisition, the input layer is designed with 13 nodes. According to the theory of a three-layer neural network, the number of hidden layer nodes can be set as 2*k* + 1, resulting in 27 nodes to approximate any nonlinear mapping. Since there are four detection targets to be identified, the output layer is set with four nodes. The designed neural network controller is shown in Figure 15.

After setting the number of nodes in each layer of the BP neural network, 459 connection weights and 31 thresholds can be calculated, resulting in an individual encoding length of 490 for determining fitting types. Once the genetic algorithm’s population size is determined, the fitness function must be defined. The fitness value F of each individual in the population is calculated by using the initial weights and thresholds corresponding to the individual’s encoding in the BP neural network for prediction and then computing the absolute error between the output and expected data.

(24) $$F=\left(\sum_{k=1}^{n}\left|C_{i}-T_{k}\right|\right)$$

Here, *n* denotes the number of network output nodes; the network’s actual output data are represented by a specific symbol $C_{k}=(c_{1},c_{2},c_{3},\cdots ,c_{q})$, while the target output data are indicated by $Tk=(y_{1}^{k},y_{2}^{k},y_{3}^{k},\cdots ,y_{p}^{k})$. During the GA-optimised BP neural network process, the selection operation employs roulette wheel selection, a fitness-proportionate strategy. The selection probability for individual *i* is calculated using the following formula:

(25) $$p_{i}=\frac{F_{i}}{\sum_{i=1}^{n}F_{i}}$$

Here, *F_i_* represents the fitness value of the *i*-th individual, and *n* is the population size. In this study, we use real-number encoding for the genetic algorithm population. Consequently, we employ single-point crossover, suitable for real-number encoding. Specifically, during crossover, the *k*-th and *l*-th individuals exchange genetic segments at gene position *j*.

(26) $$\left\{\begin{array}{l} a_{kj}=a_{kj}(1-b)+a_{lj}b\\ a_{lj}=a_{kj}(1-b)+a_{kj}b \end{array}\right.\begin{array}{cc} & b\in [0,1] \end{array}$$

Here, *b* is a random number between (0, 1). In the genetic algorithm, mutation is a key operation that helps maintain the diversity of the population dataset by generating new population data. Specifically, this operation is performed at position *j* of a randomly selected individual *i* from the parent population.

(27) $$a_{ij}=\left\{\begin{array}{l} a_{ij}+(a_{ij}-a_{\max})\cdot r_{2}{\left(1-\frac{g}{G_{\max}}\right)}^{2},r>0.5\\ a_{ij}+(a_{\min}-a_{ij})\cdot r_{2}{\left(1-\frac{g}{G_{\max}}\right)}^{2},r<0.5 \end{array}\right.\begin{array}{cc} & r_{2}\in [0,1] \end{array}$$

Here, *a_max_* and *a_min_* are the lower and upper bounds of *x*; *g* is the current iteration number; G is the maximum number of generations; and r is a random number between (0, 1). The fitness function of the new population is calculated. If the fitness meets the criteria or the maximum generations are reached, the evolution stops; otherwise, it returns to the selection phase of the genetic algorithm. Once the initial weights and thresholds optimised by the genetic algorithm (GA) meet the conditions, they are passed to the BP neural network for predictive computation.

## 5. Experimental Results Verification

### 5.1. Physical Experiment

**Figure 16:**
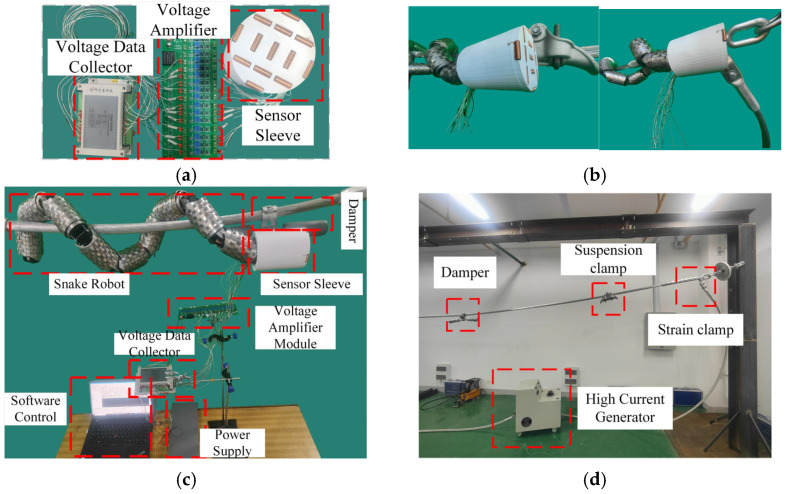
The experimental signal, collection devices, experimental environment, and the detection process. (**a**) Sensor and voltage amplifier data acquisition module; (**b**) the detection of strain clamps and suspension clamps; (**c**) physical experiment setup; (**d**) experimental environment.

As shown in Figure 16a, the fitting type detection system consists of a sensor sleeve, a differential amplifier module, and a DAM-3055N voltage data collector (Beijing Altai Technology Development Co., Ltd., Beijing, China). The electromagnetic sensor array is mounted at the head joint of the snake robot. A cylindrical sleeve was fabricated using 3D printing technology, embedding 13 one-dimensional electromagnetic sensors. Since the raw signals from the sensors are weak (only a few millivolts), they are filtered and differentially amplified before being connected to the DAM-3055N voltage data collector for full-sample real-time voltage signal acquisition.

Experimental setup is shown in Figure 16b and includes the ZYSLQ-1000, a high-current generator which provides a 50 Hz, 309 A alternating current to generate a magnetic field similar to that of a high-voltage transmission line. In the experiment, LGJ-400/50 steel-core aluminium stranded wire is used (Henan Yulong Cable Co., Ltd., Zhengzhou, China), tensioned through a support frame to ensure full tension of the wire. The damper, suspension clamp, and tension clamp are suspended on the steel-core aluminium stranded wire. After power is applied, the steel-core aluminium stranded wire generates a magnetic field simulating the operating state of a high-voltage transmission line.

As shown in Figure 16a, the fitting type detection system is composed of a sensor sleeve, a differential amplification module, and a voltage collector. The electromagnetic sensor has a core size of φ6 × 25 mm, uses enamelled wire 0.3 mm in diameter, and has 143 turns of wire. The differential amplifier is model AD620, which has a signal filtering function, includes 16 input/output ports, and has a gain ranging from 1.5 to 10,000 times, an input signal range of 0.1 mV to 10 V, and an output signal range of −10 V to 10 V. The voltage collector is model DAM-3055N, which has 16 input ports, a voltage range of −5 V to +5 V, accuracy of 0.1%, a sampling rate of 10 MHz, and each channel can independently set the range. The collector includes a 485 communication module that can send sampling data to a computer via 485.

The electromagnetic sensor array needs to be installed at the head joint of the snake robot. For this purpose, a cylindrical sleeve was manufactured using 3D printing technology to embed 13 one-dimensional electromagnetic sensors. Since the original signal of the sensor is weak (only a few mV), it needs to be filtered and amplified by the AD620 differential amplifier before being connected to the DAM-3055N voltage to collector achieve real-time voltage signal sampling.

### 5.2. Sensor Signal Acquisition Results and Analysis

**Figure 17:**
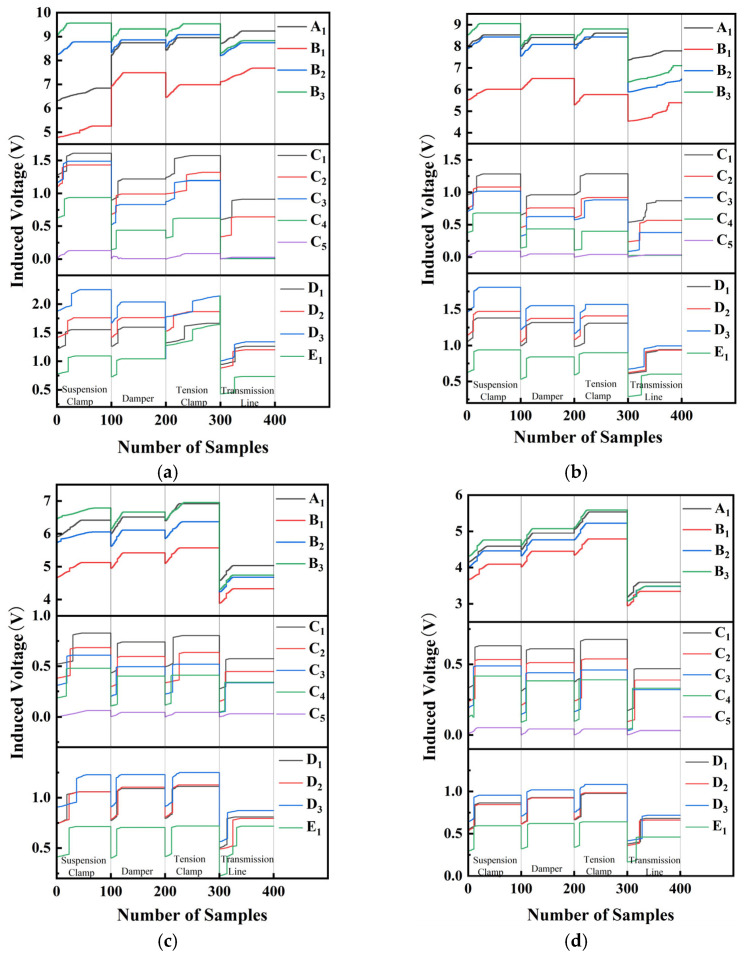
Data collected from experimental detection: (**a**) detection distance 5 cm; (**b**) detection distance 10 cm; (**c**) detection distance 15 cm; (**d**) detection distance 20 cm.

Figure 17 shows the processed raw data from the sensors, summarizing 100 sampling results for the transmission line and three types of conductor fittings (damper, suspension clamp, and tension clamp) at different detection distances.

Table 4 summarizes the relationship between the induced electromotive force signals in the B_x_ and B_y_ detection directions at a 5 cm detection distance for different electric power fittings. It can be observed that during the detection of the transmission line, the order of A_1_ and D_1_ in the A_i_, D_i_, and E_i_ series differs, allowing the A_1_ and D_1_ signals to be used as characteristic signals for the transmission line. When the conditions A_1_ > B_3_ > B_2_ > B_1_ and D_3_ > D_1_ > D_2_ > E_1_ are both met, the object can be identified as a transmission line. For the remaining electric power fittings, classification is based on the range variation trends of the B_y_ signals in the X and Y directions from the A_i_, B_i_, D_i_, and E_i_ series sensors, as well as the range variation trend of the B_x_ signal in the Y direction from the C_i_ series sensors.

(28) $$\Delta \varepsilon =\varepsilon_{\max}-\varepsilon_{\min}$$

**Figure 18:**
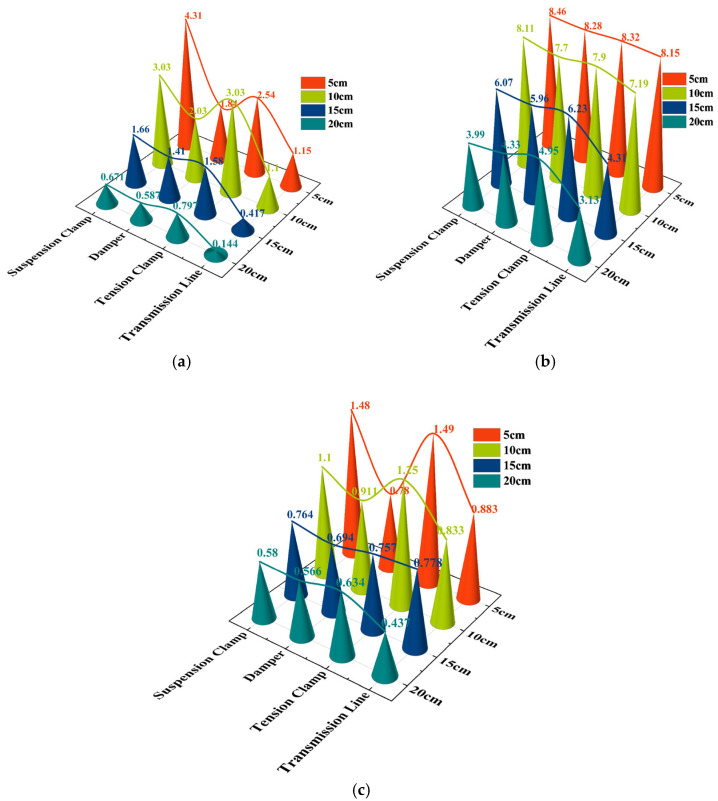
Range variation of *B_y_* and *B_x_* induced electromotive force in X and Y directions at different distances: (**a**) range variation of *B_y_* induced electromotive force in Y direction; (**b**) range variation of *B_y_* induced electromotive force in X direction; (**c**) range variation of *B_x_* induced electromotive force in Y direction.

∆*ε* represents the range of the induced voltage signal in volts (V); *ε_min_* is the minimum induced electromotive force in volts (V); *ε_max_* is the maximum induced electromotive force in volts (V). An in-depth analysis of Figure 18 reveals that the induced electromotive force (EMF) polarization detected by the induction coil sensor decreases in both X- and *Y*-axis components as the detection distance increases, and this phenomenon is highly consistent with the trend of the variation of the magnetic field strength around the fixture along the X*Y*-axis component as established in Section 3, which means that the magnetic field strength gradually decreases with the increase in the distance. However, at the same detection distance, the trend of the magnitude of the induced electric potential polarization of different fixtures remains stable, always following the pattern of ∆*ε _suspension clamp_ >* ∆*ε _tension clamp_ >* ∆*ε _damper_ >* ∆*ε _transmission line_*. This trend coincides with the pattern obtained in Section 3 based on the magnetic field strength model of the fixture, which is analysed in Table 2, and the relationship between the magnitude of the magnetic field strength poles is as follows: ∆*B_suspension clamp_ >* ∆*B_tension clamp_ >* ∆*B_damper_ >* ∆*B_transmission line_*. The experimental results not only strongly verify the accuracy of the magnetic field model established in Section 3 but also fully reflect the reasonableness of the induction coil arrangement adopted in this paper and show that the overall idea of utilizing the difference in magnetic field strength to distinguish the type of gold tools has significant validity.

### 5.3. Classification Results of the GA-BP Neural Network Algorithm

First, the transmission lines and other conductor fittings are manually classified and numbered, as shown in Table 5. Finally, the induced electromotive force data collected from each fitting are input into the neural network model optimised by the genetic algorithm. In the collected experimental data, 80% are randomly allocated to the training set, and the remaining 20% are used as the test set. In the network parameter configuration, the maximum number of training iterations is set to 5000, and the optimization target for the mean squared error is 10^−7^.

The hyperbolic tangent (tansig) transfer function is used from the input layer to the hidden layer, and a pure linear (purelin) transfer function is used from the hidden layer to the output layer, which implements an identity mapping of the neuron values, directly passing them to the next layer.

**Figure 19:**
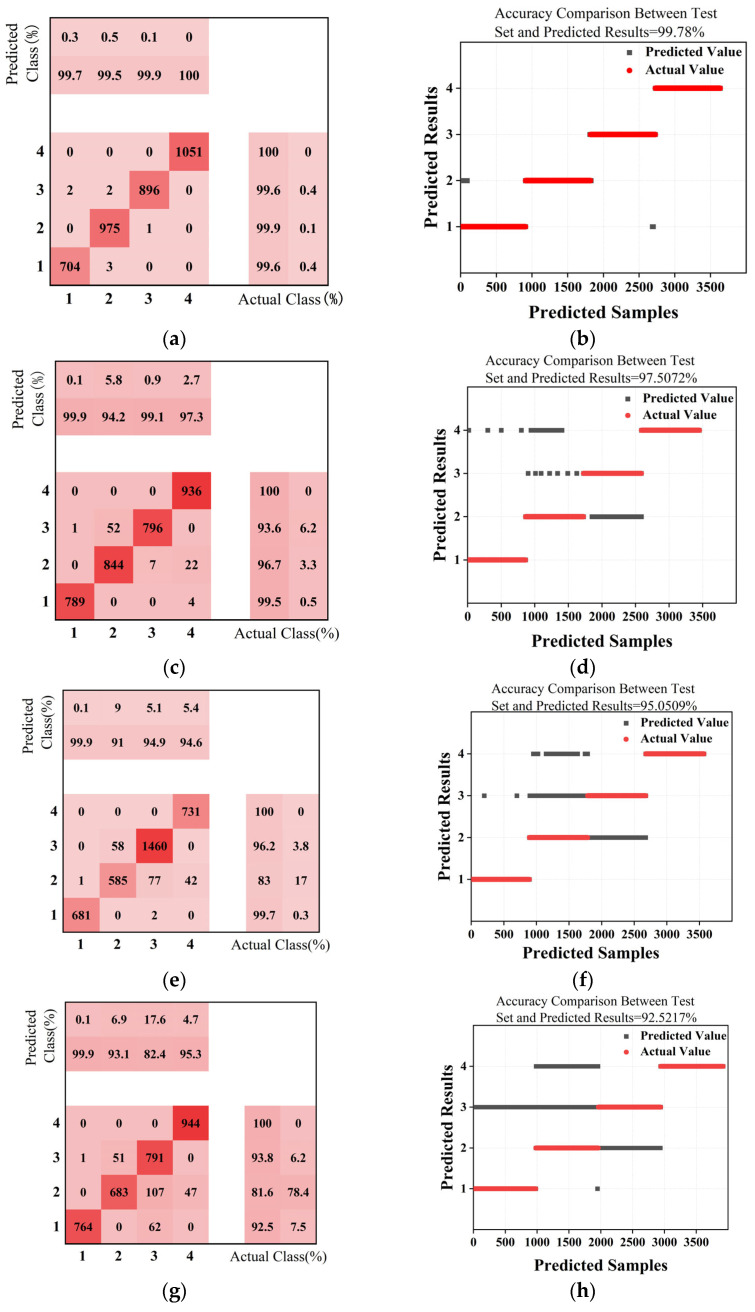
Output results: (**a**) confusion matrix for 5 cm prediction; (**b**) comparison of 5 cm prediction results; (**c**) confusion matrix for 10 cm prediction; (**d**) comparison of 10 cm prediction results; (**e**) confusion matrix for 15 cm prediction; (**f**) comparison of 15 cm prediction results; (**g**) confusion matrix for 20 cm prediction; (**h**) comparison of 20 cm prediction results.

Figure 19a presents the final predicted classification results, showcasing the algorithm’s classification outcomes at a detection distance of 5 cm. The resulting confusion matrix indicates the following:

According to Table 6, the average classification accuracy across all categories of electric power fittings reaches 99.78%. The high classification accuracy and recall rates for each category indicate a strong predictive capability for type classification. Therefore, the genetic algorithm-optimised BP neural network algorithm demonstrates good accuracy in distinguishing transmission lines from the three common types of electric power fittings.

Analysis of classification results at different detection distances reveals that the accuracy of the BP neural network, optimised by a genetic algorithm, in predicting fitting types, declines gradually with increasing detection distance. Specifically, at 5 cm, the model accuracy is as high as 99.8%; it decreases slightly to 97.5% at 10 cm, further to 95.0% at 15 cm, and then to 92.5% at 20 cm. This trend aligns with the magnetic field model around fittings presented in Section 3 of this paper. According to the model, the variation trend of magnetic field strength along the X and Y axes shows that within 25 cm of the transmission line, the differences in magnetic field strength changes around different fitting types are the most significant, and these differences gradually decrease beyond this range. This reduction in magnetic field variation is directly reflected in the prediction results of the BP neural network as a decrease in detection accuracy.

From the average accuracy perspective, the model maintains an average accuracy of over 95% within the 5 cm to 10 cm detection range. However, in the 15 cm to 20 cm range, the average accuracy falls below 95%, especially at 20 cm, where it is only 92.5%. This significant change in accuracy indicates that the model’s optimal detection range is between 0 cm and 10 cm. This conclusion is highly consistent with the optimal detection range obtained through particle swarm optimization (PSO) in Section 4.2, thereby strongly proving the effectiveness of the method used in this study to eliminate position disturbances and improve detection accuracy by employing PSO.

Overall, although the model’s recognition accuracy remains above 92% within the 5 cm to 20 cm detection range, this result fully confirms the effectiveness of the transmission line fitting type classification detection method based on electromagnetic induction coils proposed in this study. The high accuracy is partly attributed to precise magnetic field modelling and the determination of the induction coil sensor installation position using PSO. Meanwhile, within closer detection ranges, even with some changes in detection position, the model can still maintain high accuracy. This further highlights the effectiveness of the genetic algorithm in optimizing the BP neural network, giving it a certain degree of robustness.

To highlight the significant advantages of the inductive coil sensor proposed in this paper for identifying fittings in weakly visual or blind environments, we introduce a related study that thoroughly investigated the detection of vibration dampers using vision-based methods. Table 7 illustrates the recognition accuracy under varying light conditions. The study reveals that as the detection distance decreases, vision-recognition cameras must constantly refocus to capture images of the dampers. Despite this effort, the accuracy remains at 70%. Furthermore, Table 7 demonstrates that light intensity substantially impacts visual recognition accuracy. During midday, when strong light causes overexposure, the recognition accuracy drops significantly. Although this study focuses solely on the visual recognition of vibration dampers, it indirectly underscores the superior performance of our proposed method in terms of detection accuracy. In contrast to vision-based approaches, our method utilizing inductive coil sensors does not rely on or suffer from lighting conditions. Within the critical close-range distance of 5–20 cm from the transmission line, the recognition accuracy of our method consistently remains above 92%. This clearly demonstrates its superiority in the field of fitting-type detection, making it more practical and reliable in weakly or non-visual environments. Consequently, our approach offers a novel and efficient solution for relevant detection tasks.

To validate the robustness of the transmission line fitting type identification method based on the genetic algorithm-optimised BP neural network (GA-BP) proposed in this paper, experiments were conducted at four gradient detection distances ranging from 5 cm to 20 cm. The experimental results tripartitely verify the reliability of the method: (1) confirming the effectiveness of the inductive coil sensor-based identification approach; (2) validating the rationality of the magnetic field distribution model constructed from cross-sectional shape differences of fittings and the sensor layout scheme based on singular value decomposition (SVD) of the position matrix; (3) fully demonstrating the strong robustness of GA-BP to inductive coil signals.

Notably, despite the interference from the ferromagnetic materials of the snake-shaped robot on the transmission line’s magnetic field, the experimental data reveal a counterintuitive phenomenon: the identification accuracy at 5 cm (98.7%) is significantly higher than those at other distances (10 cm: 96.3%; 15 cm: 94.1%; 20 cm: 92.5%). This outcome verifies the dual anti-interference capabilities of the GA-BP neural network: (1) adaptive suppression of interference from nearby ferromagnetic materials; (2) robustness against magnetic field intensity attenuation caused by varying detection distances. The method maintains an identification accuracy above 92% as detection distance increases and background magnetic field interference intensifies, demonstrating excellent engineering practicability.

This robustness stems from the dual advantages of the GA-BP algorithm: the genetic algorithm-optimised weight initialization effectively avoids the local optimality problem of BP networks, while the multi-layer perceptron structure enhances the feature extraction capability for nonlinear magnetic field signals.

## 6. Conclusions and Future Work

In scenarios with blind or poor visibility, visual sensors struggle to effectively identify fittings on high-voltage transmission lines. To tackle this, this paper proposes using inductive coil sensors to detect the differences in magnetic field strength around fittings for type differentiation. The research process is as follows:

A magnetic field distribution model, considering the impact of the fitting detection cross-section, was established by integrating the Dodd–Deeds eddy current model with the cross-sectional characteristic function of wire fittings. Via this model, the magnetic field strength distribution of vibration dampers, tension clamps, and suspension clamps at the detection cross-section was theoretically compared, with the variation trend in magnetic field strength serving as the classification feature. Results showed the magnetic field difference order as ∆*B_suspension clamp_ >* ∆*B_tension clamp_ >* ∆*B_damper_ >* ∆*B_transmission line_*.

Based on the variation trend of magnetic field strength along the X and Y axes, the initial arrangement of inductive coil sensors along these axes was determined. For each sensor spacing, the concept of the condition number was introduced, and the impact of detection position on fitting classification accuracy was analysed using singular value decomposition (SVD). An improved particle swarm optimization (PSO) algorithm with adaptive inertia weight adjustment was then employed to derive the optimal detection position matrix, reducing the influence of detection position on classification results. Considering the snake robot’s structural characteristics, the distribution of inductive coil sensors and the optimal detection range (within 0–12.5 cm) were finally determined.

A sensor distribution capable of detecting magnetic field variation signals from different fittings was constructed. The inductive coil sensors detected the magnetic field strength differences around fittings, and a BP neural network optimised by a genetic algorithm was used for fitting classification. Experimental results showed that at detection distances of 5 cm, 10 cm, 15 cm, and 20 cm, the model accuracy was 99.8%, 97.5%, 95.0%, and 92.5%, respectively. This aligns with the magnetic field model in Section 3, where within 25 cm of the transmission line, the variation in magnetic field strength around different fittings is most significant. Beyond this range, the variation decreases, reducing detection accuracy.

From the average accuracy perspective, within the 5–10 cm detection range, the average accuracy remained above 95%; in the 15–20 cm range, it fell below 95%, especially at 20 cm, where it was only 92.5%. This indicates the model’s optimal detection range is 0–10 cm, consistent with the PSO-optimised range in Section 4.2. This demonstrates PSO’s effectiveness in eliminating detection position disturbances and improving accuracy.

Overall, within the 5–20 cm detection range, the model’s recognition accuracy remained above 92%, confirming the effectiveness of the electromagnetic induction coil-based transmission line fitting classification method. The high accuracy is partly due to precise magnetic field modelling and the PSO-determined inductive coil sensor installation position. Within shorter ranges, even with slight position changes, the model maintains high accuracy, highlighting the genetic algorithm’s effectiveness in optimizing the BP neural network and enhancing its robustness.

In conclusion, the detection method and optimization algorithms used in this study offer significant advantages for transmission line fitting classification, achieving high-precision detection and providing robust support for related research and applications.

In follow-up research, this project has multi-aspect expansion potential:

This study plans to deepen and expand from multiple dimensions in the future. On the one hand, inductive coil sensors will be used to broaden the detection scope, enabling the detection of more types of transmission line fittings and applying it to the pose detection of serpentine inspection robots. The possibility of electromagnetic sensors identifying robot joint poses and serving as motion control feedback will be explored. On the other hand, the existing fitting type differentiation method will be integrated with other sensors to enhance recognition accuracy under complex conditions. In terms of algorithms and models, advanced deep learning models like CNN will be explored to optimise magnetic signal processing. To address the limitation of not considering complex working conditions interference, disturbance experiments will be designed to assess the impact. Technologies like multi-sensor fusion will be introduced to improve system stability and adaptability. The related content will be refined in subsequent papers to strengthen the application value and technical level of the research in the field of transmission line inspection and fitting detection.

## Figures and Tables

**Table 1:** Comparison of common sensor types.

Sensor Type	Environmental Adaptability	Best Distance for Identifying Obstacles	Accuracy Rate	Main Limitations
Industrial Camera [14]	Visual	1.5 m	70%	Depends on lighting, cannot work blindly
LiDAR [23]	Visual	16 m	85.5%	Often combined with a camera, high cost
Non-contact Inductive Coil	Non-visual	5 cm	99.8%	All-weather operation, independent of light

**Table 2:** Magnetic induction intensity distribution polarities in the X and Y directions.

Magnetic Field Density	Direction of Change	Transmission Line *B*_1_ (mT)	Damper *B*_2_ (mT)	Tension Clamp *B*_3_ (mT)	Suspension Clamp *B*_4_ (mT)
B_x_	X	742.15	742.4	4434.26	1426.98
	Y	664.68	1183.55	3550	3657
B_y_	X	845	3073.22	7141.79	4427.97
	Y	742	2787.46	4951.84	3296.44

**Table 3:** Basic parameter table for electric power fittings simulation.

Types of Electric Power Fittings	Basic Parameter
damper	Model: FD-6 Diameter: 70 mm Spacing: 75 mm
tension clamp	Model: NL-4 Envelope thickness: 10 mm Line distance: 120 mm
suspension clamp	Model: AXS 1880 Coupling thickness: 18 mm Line spacing: 110 mm

**Table 4:** Relationship of induced electromotive force magnitude in detection results.

5 cm	Suspension Clamp	Damper	Tension Clamp	Transmission Line
B_y_	B_3_ > B_2_ > B_1_ B_3_ > B_2_ > A_1_ > B_1_	B_3_ > B_2_ > B_1_ B_3_ > B_2_ > A_1_ > B_1_	B_3_ > B_2_ > B_1_ B_3_ > B_2_ > A_1_ > B_1_	B_3_ > B_2_ > B_1_ A_1_ > B_3_ > B_2_ > B_1_
B_y_	D_3_ > D_2_ > D_1_ > E_1_	D_3_ > D_2_ > D_1_ > E_1_	D_3_ > D_2_ > D_1_ > E_1_	D_3_ > D_1_ > D_2_ > E_1_
B_x_	C_1_ > C_3_ > C_2_ > C_4_ > C_5_	C_1_ > C_3_ > C_2_ > C_4_ > C_5_	C_1_ > C_3_ > C_2_ > C_4_ > C_5_	C_1_ > C_3_ > C_2_ > C_4_ > C_5_

**Table 5:** State classification.

State	Damper	Tension Clamp	Suspension Clamp	Transmission Line
Output Node Representation	1	2	3	4

**Table 6:** GA-BP classification algorithm results at 5 cm detection distance.

Category	Accuracy	Recall	F1 Score
1	99.7%	99.7%	99.72%
2	99.6%	99.5%	99.55%
3	99.9%	99.9%	99.9%
4	100%	100%	100%
Accuracy	99.78%		

**Table 7:** Visual recognition accuracy of vibration dampers under the influence of light [14].

Experiment Time	Number of Photos Captured	Recognition Accuracy
Morning	1000	70%
Noon	1000	67.2%
Evening	1000	71%

## Data Availability

Data are contained within this article.
